# Acute FPIES and DIES: is a G lacking?

**DOI:** 10.3389/fped.2023.1185196

**Published:** 2023-06-20

**Authors:** Elio Novembre, Mattia Giovannini, Francesco Catamerò, Giulia Liccioli, Lucrezia Sarti, Simona Barni, Francesca Mori

**Affiliations:** ^1^ Department of Health Sciences, University of Florence, Florence, Italy; ^2^ Allergy Unit, Meyer Children’s Hospital IRCCS, Florence, Italy

**Keywords:** definition, FPIES, rare allergic diseases, DIES, pediatrics

Food-Protein Induced Enterocolitis Syndrome (FPIES) is a clinical entity that, in the last years, has become significantly more relevant; it has been the focus of an increasing number of publications in the scientific community. The first clinical reports suggestive of chronic FPIES are from 1960 to 1970, and they described the main presenting feature as protracted diarrhea in newborns (1–3).

In 1978 (4) diagnostic criteria for these children were proposed: (1) symptoms presenting at less than two months of age; (2) while receiving the causative formula, the infant has watery stools with mucus, blood, and leukocytes, and a peripheral polymorphonuclear leukocytosis; (3) diarrhea ceases and normal growth resumes when the offending antigen is eliminated; (4) the response to a challenge meets the criteria described.

Later, in 1986, these criteria were modified to incorporate acute symptoms like vomiting: (1) disappearance of the symptoms of vomiting and diarrhea and of diagnostic findings in the stool (blood and leucocytes) after all antigens are removed from the diet; (2) no other cause for the colitis is demonstrable; (3) symptoms do not recur, and weight gain is normal for one month on a low-antigen formula, such as breast milk or casein hydrolysate formula, as the only dietary source; (4) a challenge with milk or soy formula, or other offending food antigens, reproduces symptoms (5).

Sicherer, in 1998, included in the criteria of repetitive vomiting, the main symptom of acute FPIES in subjects aged younger than 9 months at initial diagnosis, and coined FPIES as a syndrome (6).

In 2013, Miceli Sopo (7) proposed to increase the age of presentation to less than two years and introduced a 2–4 hs latency between exposure to the incriminating food and elicitation of repetitive and important vomiting, pallor, hyporeactivity, and lethargy.

In 2015, Leonard divided the criteria for acute reactions into major and minor criteria and proposed that the criteria should not include an age limit for the age of onset. Repetitive vomiting or protracted diarrhea (1) was considered a major criterion, together with the absence of cutaneous and respiratory symptoms suggestive of an IgE-mediated allergy (2), removal of causative food results in resolution of symptoms (3), and re-exposure or a food challenge eliciting the typical symptoms (4). Minor criteria were added: hypotension (1), lethargy, pallor or hypotonia (2), negative skin-prick test and undetectable specific IgE level (3), absence of fever or hypothermia (<36°C) (4) (8).

In 2017, Nowak-Węgrzyn et al. (9) published the International Consensus Guidelines for the diagnosis and management of FPIES that, differently from previous authors, identified one major criterion and nine minor criteria, emphasizing the symptom of vomiting. The acute FPIES diagnosis is made if the major criterion and at least 3 minor criteria are met. Repetitive vomiting in the 1–4 h/s period after ingesting the suspected drug and the absence of classic IgE-mediated allergic skin or respiratory symptoms was considered the only major criterion. If only a single episode has occurred, a diagnostic Oral Food Challenge (OFC) should be strongly considered to confirm the diagnosis, especially because viral gastroenteritis is so common in this age group. Minor criteria were identified as: a second (or more) episode of repetitive vomiting after eating the same suspect food (1), repetitive vomiting episode 1–4 h/s after eating a different food (2), extreme lethargy with any suspected reaction (3), marked pallor with any suspected reaction (4), need for emergency department visit with any suspected reaction (5), need for intravenous fluid support with any suspected reaction (6), diarrhea in 24 hs (usually 5–10 hs) (7), hypotension (8), hypothermia (9).

In 2021, Vazquez-Ortiz and Infante proposed to add crampy abdominal pain, nausea (if vomiting is absent), and an increase in absolute neutrophil count >1,500/mm^3^ with normalization within 24 hs in the minor criteria to improve diagnostic accuracy (10).

Chronic FPIES is not as characterized as acute FPIES. It develops with regular/repeated ingestion of the triggering food (e.g., feeding an infant on cow's milk- or soy-based formula), presenting e.g., as chronic/intermittent emesis, watery diarrhea, and failure to thrive. Severe chronic FPIES can lead to dehydration and shock, potentially requiring bowel rest and intravenous fluids. However, for chronic FPIES, a clear set of criteria has not been defined yet. In 2015, Consensus Guidelines (9) defined the resolution of the symptoms within 3–10 days after the elimination of the offending food(s). Acute recurrence of symptoms when the food is reintroduced, the onset of vomiting in 1–4 h/s and diarrhea in 24 hs (usually lasting 5–10 hs) were reported as the most important criterion for chronic FPIES diagnosis. However, without confirmatory challenge, the diagnosis of chronic FPIES remains presumptive.

Hypoalbuminemia and poor weight gain can predict chronic cow’s milk induced FPIES in young infants with chronic gastrointestinal symptoms (9).

Vomiting is the most common symptom of pediatric FPIES in cohort studies (11, 12) meanwhile, in the adult form case series of FPIES, abdominal pain and diarrhea were more frequently reported, but vomiting is still often present (13) (Table 1).

**Table 1:** Most common symptoms of food-protein induced enterocolitis syndrome in several published studies in children.

Studies in children	Vomiting	Diarrhea	Lethargy	Pallor	Abdominal pain	Hypotension
Mehr et al. (*n*/%) [*n* = 35; 66 episodes] (21)	66 (100)	16 (24)	55 (85)	44 (67)	–	–
Katz et al. (*n*/%) [*n* = 44; cow's milk] (22)	44 (100)	11 (25)	34 (77)	6 (14)	–	–
Sopo et al. (*n*/%) [*n* = 66] (23)	65 (98)	36 (54)	–	53 (80)	–	51 (77)
Ruiz-Garcia et al. (*n*/%) [*n* = 16] (24)	16 (100)	9 (56)	4 (25)	3 (19)	–	–
Ludman et al. (*n*/%) [*n* = 50 acute; *n* = 54 chronic] (25)	44 (81)	20 (37)	9 (17)	8 (15)	3 (6)	–
Caubet et al. (*n*/%) [*n* = 74 positive OFCs] (26)	70 (96)	5 (7)	5 (7)	–	59 (80)	14 (19)
Barni et al. (*n*/%) [*n* = 51] (27)	51 (100)	10 (20)	40 (78)	38 (75)	–	3 (6)
Mehr et al. (*n*/%) [*n* = 230] (28)	230 (100)	81 (35)	172 (75)	179 (78)	–	36 (16)
Ruffner et al. (*n*/%) [*n* = 462] (29)	462 (100)	254 (55)	23 (5)	23 (5)	–	23 (5)

OFC, oral food challenge.

So far, there are no specific laboratory tests for the diagnosis of this disease, and only OFCs have shown to be useful in its diagnosis (14). Also, compared to IgE-mediated diseases, adrenaline does not represent the first line of treatment, not showing an improvement in patients’ symptoms or disease resolution. Ondansetron (a serotonin 5-HT3 receptor antagonist used to treat nausea and vomiting, often after chemotherapy) is reported as a successful treatment for vomiting, abdominal pain, and lethargy during FPIES (15, 16).

Gastric involvement is therefore considered essential for the diagnosis of a possible acute FPIES but has not been included in the acronym, which is why we think that the term Food-Protein Induced Gastro Enterocolitis Syndrome (FPIGES) may be considered by the scientific community.

In 2014, Novembre et al. first described an amoxicillin-induced adverse reaction with the same characteristics as FPIES, named drug-induced enterocolitis syndrome (DIES) (17).

In 2019, the diagnostic criteria for patients with possible DIES were proposed (18).

DIES shares with FPIES the clinical presentation of vomiting in the 1–4 h/s period after ingestion of the suspected drug and the absence of classic IgE-mediated allergic skin or respiratory symptoms as the major diagnostic criterion. Minor criteria were identified: a second episode of repetitive vomiting after ingestion of the same drug (1), repetitive vomiting episode 1–4 h/s after ingestion of a different drug (2), extreme lethargy (3), marked pallor (4), need for emergency department visit (5), need for intravenous fluid support (6), diarrhea in 24 hs (usually 5–10 hs) after ingested drug (7), hypotension (8), hypothermia (9). The diagnosis of DIES requires that a patient meet the major criteria and at least 3 minor criteria. If only a single episode has occurred, a diagnostic OFC should be strongly considered to confirm the diagnosis.

In the reported cases of DIES, vomiting in the 1–4 h/s period after ingestion of the suspected drug is the only symptom always present (19, 20). Gastric involvement is therefore considered crucial for the diagnosis of possible DIES but has not been included in the acronym, which is why we think that the term Drug-induced Gastro Enterocolitis Syndrome (DIGES) may be considered by the scientific community.

In conclusion, in our opinion, the terms FPIGES and DIGES, underlining the classic gastric involvement in FPIES and DIES, may be considered by the scientific community. This may lead to a better description of these clinical entities to correctly recognize these underdiagnosed syndromes and guide their treatment.
